# What about the Use of Ice Cream as a Supplementary Diet in Chronic Kidney Disease? A Case–Control Study

**DOI:** 10.3390/medsci12020022

**Published:** 2024-04-23

**Authors:** Daniela Metro, Francesco Corallo, Davide Cardile, Guido Gembillo, Luigi Manasseri, Domenico Santoro, Martina Buda, Rocco Salvatore Calabrò, Lilla Bonanno

**Affiliations:** 1 Department of Biomedical and Dental Sciences and Morphofunctional Imaging, University of Messina, 98122 Messina, Italy; daniela.metro@unime.it (D.M.); luigi.manasseri@unime.it (L.M.); 2 IRCCS Centro Neurolesi Bonino-Pulejo, S.S. 113 Via Palermo, C.da Casazza, 98124 Messina, Italy; francesco.corallo@irccsme.it (F.C.); davide.cardile@irccsme.it (D.C.); lilla.bonanno@irccsme.it (L.B.); 3 Department of Clinical and Experimental Medicine, University of Messina, 98125 Messina, Italy; guido.gembillo@unime.it; 4 Department Oncological D.A.I., UOC of General Surgery—Oncology, 98125 Messina, Italybudamartina3@gmail.com (M.B.)

**Keywords:** dietary regimens, nutritional and healthy substances, mental health, chronic kidney disease

## Abstract

Maintaining a healthy lifestyle can extend life expectancy and improve a person’s health status. In addition to physical activity and bad habits related to smoking and alcohol, diet is also a determining factor. Following a healthy diet pattern over time and supporting a healthy body weight contributes to reducing the risk of developing more severe complications associated with very common diseases such as chronic kidney disease (CKD), diabetes, or cardiovascular diseases. The 2015–2020 Dietary Guidelines for Americans promote the adoption of fat-free or low-fat diets and discourage the consumption of foods with added sugar and solid fats, such as ice creams and other frozen desserts. On the other hand, ice cream, from a nutritional and healthy point of view, can be considered a possible food choice, due to its greater palatability and high nutritional content, but its consumption must be scheduled in a balanced diet. In this retrospective study, 36 patients with chronic renal failure were enrolled. Two different diets were proposed (A and B). In Diet B, lemon sorbet was added twice a week as an alternative food to replace fruit or snacks making the diet more varied and palatable. Nutritional status and biohumoral, immunological, and blood parameters were evaluated after 6 months. A statistical analysis shows a significant inter-group difference in creatinine and azotemia between T0 and T1. Intra-group significant differences were found in lymphocytes (*p* = 0.005) and azotemia (*p* < 0.001) in Diet A, and in azotemia (*p* < 0.001) and transferrin (*p* < 0.001) in Diet B. The results indicated that ice cream represented a good alternative food in both groups of patients regarding nutritional values and patient satisfaction. Furthermore, the treatment with ice cream allowed for better control of azotemia, maintaining stable levels even in patients with advanced CKD. This study concludes that ice cream could exert beneficial effects in addition to CKD patients’ dietary regimens.

## 1. Introduction

In contemporary society, overeating, sedentariness, and the rate of obesity and overweight have increased disproportionately. This is why today, more than ever, health and well-being are strongly determined by lifestyle choices and dietary habits. The importance of adopting a balanced lifestyle and maintaining a healthy diet cannot be overstated, particularly in the context of chronic diseases such as chronic kidney disease (CKD). Lifestyle encompasses myriad factors including physical activity levels, smoking habits, alcohol consumption, stress management, and sleep patterns. Engaging in regular physical activity has been associated with numerous health benefits, including improved cardiovascular health, weight management, and enhanced mental well-being. Conversely, sedentary behavior has been linked to an increased risk of developing various chronic conditions, including CKD. In tandem with lifestyle, dietary habits exert a profound influence on health outcomes [1]. A well-balanced diet rich in fruits, vegetables, whole grains, and lean proteins is essential for maintaining optimal health. On the other hand, diets high in processed foods, saturated fats, sodium, and sugar have been implicated in the development and progression of CKD, as well as other comorbidities such as hypertension and diabetes mellitus. This gives a measure of how useful dietary and lifestyle habits can be in promoting health and managing CKD, considering the specificities of each patient. Individuals diagnosed with CKD face unique challenges regarding lifestyle and dietary management. Given the impaired renal function characteristic of CKD, patients must adhere to dietary restrictions to prevent further kidney damage and manage associated complications such as electrolyte imbalances and fluid overload. Key dietary modifications typically include limiting sodium intake, restricting phosphorus and potassium-rich foods, and monitoring protein consumption [2]. Furthermore, CKD patients may benefit from dietary interventions tailored to their specific stage of kidney disease. For instance, individuals in the early stages of CKD may focus on slowing disease progression through blood pressure control and protein restriction, whereas those in advanced stages may require more aggressive dietary modifications, including potassium and phosphorus restriction, to mitigate symptoms and complications associated with renal failure [3].

Diet, however, constitutes one of the therapeutic interventions that is most poorly followed in a literal way by the patient. In addition to this, very often, it is difficult to maintain the above prescription for prolonged periods of time. After an initial improvement (in terms of weight or health, for example) patients make it difficult to maintain the dietary regimen for excessively prolonged periods as they tend to become frustrated and unwilling to give up opportunities to meet with significant others. This is especially the case in patients with CKD in whom diet-related difficulties are compounded by a whole range of disease-related challenges [4].

Healthcare professionals should provide comprehensive education and support to CKD patients in order to facilitate adherence to lifestyle and dietary recommendations [5].

Since, as mentioned, lifestyle and dietary factors play a crucial role in promoting health and optimizing chronic conditions in CKD, creating a patient-friendly diet could improve patient adherence to treatment, enhancing the overall satisfaction and quality of life, and reducing morbidity and mortality associated with the drop-out [6]. For all these reasons, in this study, ice cream—a frozen dessert known and consumed worldwide—was considered a valid option to make a diet more palatable for these patients.

The 2015–2020 Dietary Guidelines for Americans promote the adoption of a fat-free or low-fat diet and discourage the consumption of foods with added sugar and solid fats, such as ice creams and other frozen desserts [7]. The belief that ice cream is an unhealthy product can be reverted by analyzing its nutritional profile, which is rich in well-defined beneficial elements. Ice cream’s benefits can be relevant, especially in the elderly, in subjects at risk of malnutrition, or in people whose health conditions are limiting their nutrient intake in regular daily meals [8]. Following a healthy diet pattern over time and supporting a healthy body weight contributes to reducing the risk of developing more severe complications associated with very common diseases such as diabetes, cardiovascular diseases, or chronic kidney disease (CKD). Individualized nutrition education demonstrated long-term positive effects on nutritional status and mental health, reductions in hospital admissions, and increases in survival among patients with advanced CKD [3].

At the same time, it is of pivotal importance to find attractive food to avoid low adherence to a balanced alimentary regimen. Low adherence to CKD dietary recommendations is associated with impaired renal function, the worsening of dyslipidemia, and inflammation [9]. In the diet of nephropathic patients (low in protein, sodium, potassium, and phosphorus), ice cream has long been banned. However, as these patients are on a restricted diet for extended periods, ice cream could be a valid and enjoyable alternative to other foods during non-acute and moderately severe periods, making the diet less monotonous and more rewarding. Since nephropathic patients on conservative therapy must follow a rather strict low-protein diet with limited sodium, phosphorus, and potassium intake based on anthropometric sides, ice cream offers a wide range of uses in healthy and diseased conditions; this food can be helpful in improving diet adherence, thanks to its considerable nutritional value and palatability. A prospective hospital-based study demonstrated that the consumption of ice cream in a balanced diet improves the quality of life for malnourished cancer patients [10]. The flavor characteristics of ice cream improve its interest compared to other foods. The main components of this ready-to-eat food are proteins, fats, carbohydrates, minerals, vitamins, fibers, and water [11]. According to its composition, ice cream is mainly divided into the following classes [12]: milk-based ice cream, fruit-based ice cream and sorbets, frozen ice pops, ice cream with vegetable fats, and yogurt-based ice cream (Table 1).

Milk-based ice cream’s main components are milk, sugar, and eggs, with the addition of flavoring ingredients (e.g., chocolate, cocoa, coffee, nougat, hazelnuts, and liqueurs).

Fruit-based ice creams and sorbets, consisting mainly of fresh or frozen fruit, sugar, and water, have a caloric value lower than milk-based ice cream due to the reduced presence of proteins and lipids; nevertheless, they present greater glucose contents [13]. The only fruit ice creams based on water are strawberry ice cream and lemon ice cream. Frozen ice pops have the least nutritional content with respect to the above-mentioned classes and could be an ideal tool for preventing dehydration.

From a nutritional point of view, ice creams containing vegetable fats are recommended for their polyunsaturated fatty acids content. Moreover, ice creams could also be considered a ready-to-use food with potential beneficial health properties due to their micronutrient content. Several foods that represent an excellent source of nutrients may be wrongly identified as a source of harmful constituents [14]. For example, ice creams can be functional, and can also have a potential nutraceutical interest due to beneficial health substances. These nutrients can be added or are already contained in the ice cream preparation, especially in fruit-based ice creams [15,16]. Yogurt-based ice creams are experiencing growing interest. They can be divided into two classes: the 100% yogurt-based and the mixed yogurt–milk-based (from 20% up to 40% milk content).

Diet habits affect people’s nutritional status and health; the possibility of modifying a monotonous specific diet represents a winning strategy to guarantee patients’ adherence to balanced alimentary regimens [17,18].

In this study, the addition of ice cream into low-protein diets with sodium restrictions has been analyzed, particularly in nephropathic patients experiencing a restricted diet for a very long period. These are patients with CKD (stage 2–4) undergoing conservative treatment. From a nutritional and health point of view, ice creams can be considered a possible food choice in place of other foods with similar bromatological characteristics and offer a wide range of uses, mostly for their palatability. The aim of the study has been to improve the monotonous specific diets of CKD patients.

The optimal management of patients with chronic kidney diseases requires appropriate clinical interpretation and recognition, drug therapy, and a recommended special diet. A protein- and electrolyte-controlled diet needs to be prescribed for patients with liver disease.

The development of kidney diseases is related to noteworthy morbidity and mortality. In addition, kidney homeostasis is characterized by a complex interplay of variabilities that must be carefully balanced according to bodily response.

## 2. Methods

Our study involved 36 patients (20 women and 16 men) from the UOC of Nephrology and Dialysis, University Hospital of Messina; patients were an average age of 72 years, with chronic renal failure, neither obese nor diabetic, and undergoing conservative treatment with a normo-caloric diet (1750–1850 Kcal/day), low in proteins (42–45 g of protein/day), sodium, and a limited intake of K and P. The protein intake was evaluated according to the renal function, calculated via creatinine clearance, through the collection of urine from the last 24 h (GFR 42 mL/min, mean values).

Since dieting is often a type of intervention that is maintained over time with difficulty on the part of the subject, there is always a tendency to accommodate young and elderly patients. For this reason, diets developed at our outpatient clinics are strictly individualized and take into account each person’s dietary desires and preferences. More specifically, in the sample of patients examined, two dietary regimes, indicated with Diet A and Diet B, were proposed (Table 2). Both proposed regimens included 5 meals a day (breakfast, morning snack, lunch, afternoon snack, and dinner), and the use of extra virgin olive oil and seasonal fruits and vegetables has been recommended.

In 18 patients with Diet B, an alternative food, lemon sorbet, was added twice a week compared to Diet A, instead of fruit consumed as a snack, and with a reduction in vegetables for lunch and dinner, to make the diet more varied and more palatable. Table 3 shows the nutrient composition of fruit, vegetables, and lemon sorbet.

The nutritional status of patients was assessed via anthropometric examination (BMI) and biohumoral and immunological tests (albumin, transferrin, and lymphocyte counts; Table 4).

In addition, the blood levels of creatinine, azotemia, and electrolytes (potassium and phosphorus) were evaluated. The effects of the diet were evaluated after six months of dietary treatment (Table 4).

## 3. Statistical Analysis

A descriptive statistical analysis of sociodemographic characteristics, followed by the mean and standard deviation of the two groups, was conducted. The distribution of the data was evaluated using the Shapiro–Wilk normality test. A paired t-Student or Wilcoxon signed-rank test was used to determine if there was a difference between T0 (baseline) and T1 (after 6 months) and an unpaired t-Student or U-Mann–Whitney was used for intra-group analysis. Correlations between variables were computed with Spearman’s coefficient or Pearson correlation. Analyses were performed using an open-source R3.0 software package. A 95% confidence level was set with a 5% alpha error. Statistical significance was set at *p* < 0.05.

## 4. Results

**Figure 1:**
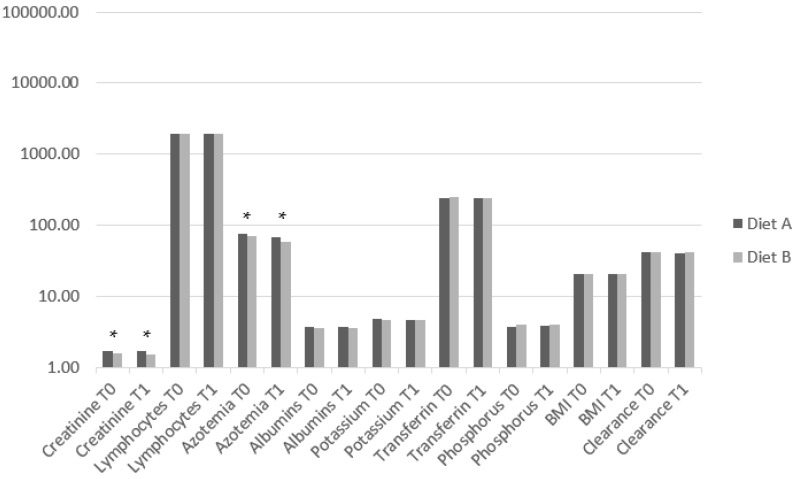
Histogram depicting the clinical data values at T0 and T1 for the two groups. Data are represented on a logarithmic scale. Legend: * significant difference.

**Figure 2:**
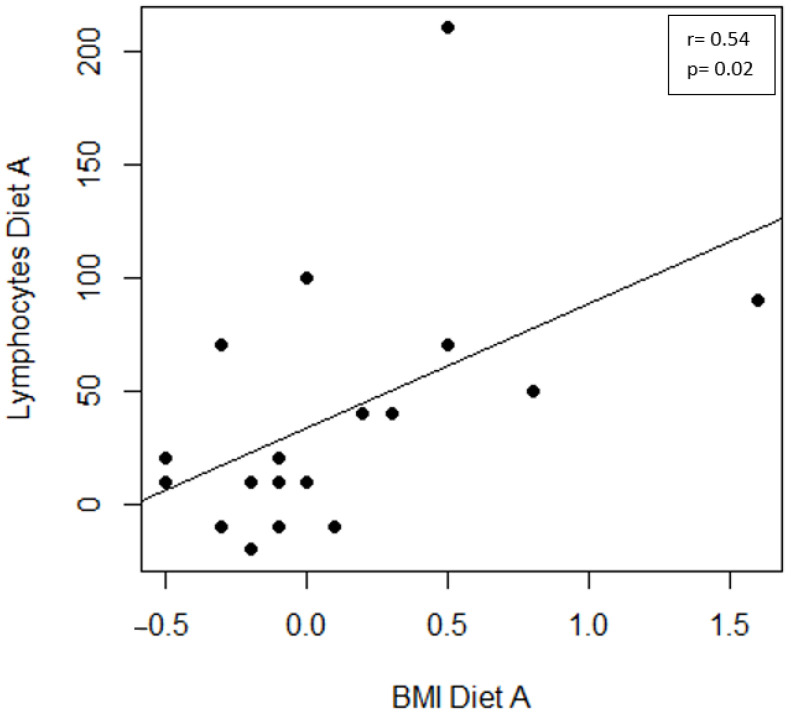
Correlation between clinical scores in the Diet A group. Scatter plot of BMI and lymphocytes.

The inter-group analysis showed a significant difference in creatinine at T0 (*p* < 0.001) and T1 (*p* < 0.001) and in azotemia at T0 (*p* < 0.001) and T1 (*p* < 0.001) (Table 4; Figure 1). The intra-group analysis highlighted significant differences between T0 and T1 in lymphocytes (*p* = 0.005) and azotemia (*p* < 0.001) in Diet A, and in azotemia (*p* < 0.001) and transferrin (*p* < 0.001) in Diet B (Table 4). A significant Spearman correlation between BMI in Diet A and lymphocytes (r = 0.54; *p* = 0.002) was found (Figure 2).

## 5. Discussion

CKD is characterized by a progressive and irreversible impairment of kidney function and is considered a growing disease. It affects a high percentage of the population; this is mainly due to the lengthening of life [19]. Nutritional dietary therapy is a significant part of the conservative management of patients with CKD, designed to complement pharmacological treatment. Its key focus is not just on reducing protein intake but also on ensuring adequate caloric intake and managing sodium, potassium, and phosphorus levels. Beyond quantity, the diet also emphasizes the quality of foods, particularly promoting plant-based foods that have beneficial effects on phosphorus metabolism and acid–base balance. The potential beneficial impacts of nutritional dietary therapy in advanced chronic kidney failure could include reduced circulating uremic toxins, decreased urea and creatinine levels, a potential reduction in inflammation, decreased intestinal permeability, a potential slowing of chronic kidney disease progression, and a reduction in intestinal dysbiosis (chronic kidney disease is marked by gut microbiota dysbiosis contributing to uremic poisoning). Moreover, advanced chronic kidney disease can be exacerbated by malnutrition. It is critical to predict, diagnose, and characterize malnutrition and monitor the response to nutritional therapy. Nutritional status should be assessed using various parameters: biochemical data (monitoring albumin, transferrin, and lymphocyte count), body mass, muscle mass, and fat mass. For this reason, many dietary strategies have been proposed to delay the progression of the disease, based on adequate nutritional interventions aimed at modifying the usual diet of patients. Dietary Nutritional Therapy (DNT) for CKD is based on limiting protein intake, reducing phosphorus intake, reducing/controlling sodium intake, controlling potassium intake, and satisfying caloric demand [20]. The proper management of DNT for CKD provides a reduction in protein intake below 0.8 g of protein/kg b.w./day, which corresponds to the recommended intake for the general healthy population [21]. Nowadays, the interest in healthy and balanced diets, in which micro and macro constituents play an important role, is growing [22,23,24,25]. In addition to ice creams that use natural ingredients (e.g., chocolate or hazelnut ice cream), other types of ice creams, consisting of potentially healthy ingredients such as W-3 fatty acids and antioxidants, have been proposed to make healthy ice cream products; ice cream consisting of a mixture of dark cocoa, hazelnuts, and green tea rich in w-3 and polyphenols and ice cream enriched with lycopene and polyphenols are two examples of such products [26].

Conventionally, standard nutritional therapy is based on an intake of 0.55–0.60 g of protein/kg b.w./day to maintain the right nitrogen balance [27].

A highly hypoproteic nutritional therapy is characterized by an intake of 0.30–0.40 g of proteins/kg b.w./day, especially of a vegetable nature [28]. In particular, in patients with glomerular filtrate (GFR) between 25–60 mL/min, the recommended protein intake is between 0.7–0.8 g/kg b.w./day.

In patients undergoing conservative therapy with GFR < 25 mL/min, the protein intake can be reduced to 0.55–0.60 g/kg b.w./day. Patients with GFR > 60 mL/min do not require protein restrictions below the recommended quota for normal subjects: 0.80–1 g/kg b.w./day. Protein metabolism is closely linked to energy intake and nitrogen demand is inversely correlated with caloric demand. Most patients with advanced CKD manage to maintain a neutral or positive nitrogen balance with 0.55–0.60 g of protein/kg b.w./day, if they have an energy intake greater than 30 Kcal/Kg b.w./day. In particular, the strict adherence to the energy prescription is 35 Kcal/Kg b.w./day in subjects under the age of 60 and 30 Kcal/Kg b.w./day in subjects over the age of 60, and it is essential for maintaining the nitrogen balance during the low-protein diet. Nitrogen balance is an essential element for maintaining a good state of nutrition and body composition. In a stable renal patient with a reduced protein intake, nitrogen balance is maintained thanks to a biological adaptation mechanism, which consists of the body’s ability to reduce protein catabolism [29,30,31,32]. During a low-protein diet, if the patient does not meet their energy needs, proteins are used for energy purposes and the nitrogen balance becomes negative with protein degradation and the loss of lean body mass. In addition, protein intake must guarantee the need for essential amino acids. In fact, an inadequate exogenous supply of essential amino acids increases endogenous nitrogen catabolism. The prescription of personalized nutritional therapy, with close nutritional monitoring, allows for the identification of anomalies and food errors and reduces the risk of malnutrition. However, protein restriction could cause malnutrition over time. Still, clinical studies have shown, through the adequate monitoring of nutrition status, that in most CKD patients, muscle mass and body composition are preserved [33]. Among the clinical prescriptions with lower adherence, diets are often found to be particularly challenging. An excessively rigid and restrictive dietary regimen can frequently lead to drop-out. This occurs because the diet significantly impacts the psychological and social life of the patient. On one hand, adopting a restrictive diet means imposing restrictions on one’s social life as well; very often, patients find themselves constantly having to decline offers and dinner invitations from friends and family, which places the individual in a state of stress and social withdrawal that is difficult to endure in the long term. On the other hand, being overweight/obese is now a cause of social stigma: when the individual undergoing the diet perceives that they are not achieving the expected improvements within the expected time frame, they may choose to change their approach or abandon the diet. This happens even when the patient compares themselves to others who have achieved more satisfying results. The patient’s motivation and sense of efficacy decrease, leading them to believe that their efforts have been in vain. Another crucial aspect is the lack of awareness regarding the need to continue with a maintenance diet once the target weight is reached. This leads to weight regain, thereby discouraging the patient from embarking on a new diet. From this perspective, the introduction of ice cream into the diet could be an ideal way to reduce the perceived rigidity of the patient’s diet. This could trigger cascading effects. The patient would become more motivated, improve their treatment adherence, and reduce the risk of drop-out. Over the long term, the possibility of indulging in ice cream occasionally could allow for less restrictive access to social life and outings with significant others, reducing the perceived sense of stress and improving overall health.

Although it may seem like a simple adjustment, a personalized diet and the incorporation of foods that the patient enjoys could be the right approach to enhance the effectiveness of these treatments. From a cognitive perspective, obesity is strongly associated with a deficit profile. The release of inflammatory cytokines in the gut correlates with an increase in the same cytokines in the brain (brain–gut axis), resulting in a decline in performance in many tasks. For CKD patients, alternative foods, such as ice cream, can make the monotonous diet more varied and more palatable.

In the diet of patients with CKD, ice cream was never allowed, even if it could be a valid and pleasant alternative to other foods, and it could make the diet more rewarding, specifically in the non-acute and medium severity period. In fact, since renal patients in conservative therapy must follow a low-protein regimen with a limited supply of Na, P, and K, it is possible to include an alternative food such as ice cream in the diet, to make the diet more varied, more palatable, and more rewarding, without altering the nutritional status and the protein and electrolyte supply. Based on the analysis of the various types of ice cream, the most suitable are certainly fruit ice creams and in particular, strawberry, lemon, or sorbets (Table 1).

## 6. Conclusions

In conclusion, it can be inferred that, although the nephropathic patients in a conservative therapy must follow a rather severe low-protein regimen, calculated on anthropometric data, considering the observed value of the glomerular filtration rate (GFR), and the blood chemistry analyses, it is possible to supplement the diet with food such as ice cream to make the diet more varied, more palatable, and more gratifying. This goal can be reached without altering the nutritional status, protein, or electrolyte supply. Furthermore, ice cream could be regarded by the patients as a moment of aggregation and socialization, which could improve their mood with positively balanced sensory stimulation.

## Figures and Tables

**Table 1:** Average composition of homemade ice cream based on a 100 g portion.

Products	Energy (Kcal)	Proteins (g)	Carbohydrates (g)	Lipids (g)	Ca (mg)	Na (mg)	P (mg)	K (mg)
Milk-based ice cream	230–180	4.5–2.0	50–20	13.7–5.0	185–60	97–1	300–1	250–3
Fruit-based ice cream	200–100	4–2.5	24.3–6.2	6.5–0	100–10	200–35	100–0	100–26
Frozen ice pop	137	-	36.5	-	-	-	-	-
Sorbet	132	0.9	34.2	-	2	18	5	42

**Table 2:** Two daily feeding proposals (Diet B includes the lemon sorbet).

	Diet A	Diet B
Breakfast	- Partially skimmed milk mL 100 - Rusks n. 2 - Sugar g 30 - Jams g 30 or honey g 30	- Partially skimmed milk mL 100 - Rusks n. 2 - Sugar g 30 - Jams g 30 or honey g 30
Snack	Rice crackers n. 3	Rice crackers n. 3
Lunch	- Fresh tomato sauce pasta g 100 - Vegetables (cooked or raw) g 150 - Extra virgin olive oil 2 tablespoons - Fresh fruits g 100	- Fresh tomato sauce pasta g 100 - Vegetables (cooked or raw) g 100 - Extra virgin olive oil 2 tablespoons - Fresh fruits g 100
Snack	- Fresh fruits g 100	- Lemon sorbet g. 100
Dinner	- Low-fat meet g 80 or fish g 120 - Vegetables (cooked or raw) g 150 - Bread g 50 - Extra virgin olive oil 2 tablespoons - Fresh fruits g 100	- Low-fat meet g 80 or fish g 120 - Vegetables (cooked or raw) g 100 - Bread g 50 - Extra virgin olive oil 2 tablespoons - Fresh fruits g 100

**Table 3:** Nutrient composition of lemon sorbet, vegetables, and fresh fruits.

	Kcal (100 g of Product)	Proteins (g)	P (mg)	Na (mg)	K (mg)
Lemon sorbet	132	0.9	5	18	42
Vegetables (average values)	15	1	32	18	210
Fresh fruits (average values)	45	0.2	12	2	120

**Table 4:** Data on the state of nutrition; values measured before and after the dietary treatment and clinical characteristics of the sample.

	Diet A	Diet B	*p*
No. patients	19	17	
Gender			
Male	9	7	
Female	10	10	
Age (mean ± SD)	72.7 ± 4.8	72.3 ± 3.7	0.76 ^+^
Lymphocytes T1	1939.44 ± 112.12	1901.11 ± 61.06	0.4 ^<^
*p*	0.005 ^±^	0.83 ^±^	
Azotemia T0	76.68 ± 2.23	69.17 ± 2.40	<0.001 ^<^
Azotemia T1	68.09 ± 1.76	59.12 ± 2.06	<0.001 ^+^
*p*	<0.001 ^+^	<0.001 ^±^	
Albumins T0	3.69 ± 0.22	3.60 ± 0.21	0.28 ^<^
Albumins T1	3.68 ± 0.18	3.60 ± 0.20	0.26 ^+^
*p*	1 ^±^	1 ^+^	
Potassium T0	4.76 ± 0.32	4.65 ± 0.50	0.59 ^<^
Potassium T1	4.69 ± 0.27	4.64 ± 0.44	0.9 ^<^
*p*	0.29 ^±^	0.82 ^+^	
Transferrin T0	241.0 ± 22.14	251.39 ± 25.79	0.1 ^<^
Transferrin T1	238.11 ± 27.58	241.11 ± 25.47	0.74 ^+^
*p*	0.77 ^±^	<0.001 ^±^	
Phosphorus T0	3.73 ± 0.58	3.98 ± 0.51	0.17 ^+^
Phosphorus T1	3.78 ± 0.48	3.97 ± 0.42	0.21 ^+^
*p*	0.46 ^+^	0.86 ^+^	
BMI T0	20.48 ± 1.59	20.49 ± 0.98	0.98 ^+^
BMI T1	20.57 ± 1.50	20.50 ± 0.97	0.87 ^+^
*p*	0.44 ^+^	0.87 ^+^	
Clearance T0	41.34 ± 3.57	41.8 ± 2.82	0.67 ^+^
Clearance T1	40.87 ± 4.05	41.69 ± 2.90	0.49 ^+^
*p*	0.48 ^+^	0.11 ^+^	

Legend: ^+^ T-Student; ^±^ Wilcoxon signed-rank test; ^<^ U-Mann–Whitney; SD = Standard Deviation.

## Data Availability

The data that support the findings of this study are available upon request from the corresponding author.
